# Synchronous Colon Carcinoma and Tuberculosis: Coincidence or Linked

**DOI:** 10.5005/jp-journals-10018-1224

**Published:** 2017-05-05

**Authors:** Seema Dayal

**Affiliations:** Department of Pathology, Uttar Pradesh University of Medical Sciences, Etawah, Uttar Pradesh, India

**Keywords:** Carcinoma colon, Intestinal tuberculosis, Mycobacterium tuberculosis.

## Abstract

**How to cite this article:** Dayal S. Synchronous Colon Carcinoma and Tuberculosis: Coincidence or Linked. Euroasian J Hepato-Gastroenterol 2017;7(1):97-98.

Dear Editor,

Carcinoma of colon and tuberculosis (TB) are common entities, but it is very uncommon to find both at same location, though sometimes both appears as differential diagnosis for each other.^1,2^ It is usually seen that carcinoma is more common in the distal large bowel and TB in the terminal ileum or ileocecal junction.^3^ Tuberculosis is a significant health problem in developing countries like India. India contributes to 1.98 million cases to the worldwide disease burden of 9.4 million cases.^4^ Pulmonary TB is the major form of TB, and intestinal TB is a rare form of extrapulmonary TB accounting for 3 to 4%. Tuberculosis in the intestine may occur as primary, secondary, and hyperplastic. Primary is extremely rare and it may occur via drinking milk contaminated with *Mycobacterium bovis,* whereas secondary TB occurs by swallowing of sputum in patients with active pulmonary TB.^3^ Tuberculosis in intestine on gross examination may be ulcerative, hyperplastic, and sclerotic. Peritoneal and mesenteric lymph nodes get infected during bac-teremic phase of TB. Intestine gets infected by retrograde lymphatic spread from mesenteric lymph nodes. Colorectal carcinoma is the most common malignancy of the gastrointestinal tract, and common locations are rectum, sigmoid colon, cecum, and ascending colon.^5^ The common variants of colorectal carcinoma are adenocar-cinoma, mucinous adenocarcinoma, signet ring cell, and small cell carcinoma. Some diseases like ulcerative colitis, Crohn’s disease, and schistosomiasis predispose to malignancy. Chronic inflammatory mucosal damage leads to metaplasia followed by dysplasia and finally neoplasia. Possibility of this phenomena was suggested by Japanese researchers.^6^ It is also known that factors that disturb host immunity, such as severe weight loss, malnutrition because of malignancy increase susceptibility to active tubercular infection. Invasion of dormant tubercular by carcinoma may lead to activation of tubercular lesion. Tumor antigens or peptides produced by tumor itself may also upset the milieu of a granuloma and allow TB bacilli to proliferate. There are studies that lead to suggestion that mycobacterium TB can be instrumental in the development of malignant disease.^7^ Similarly, other research believes any connection between active TB and malignancy is attributed to reactivation of infection in immune compromised patients suffering from cancer rather than to cause and effect relationship between infection and neoplasm.^8^

The association between intestinal TB and colon carcinoma is quite challenging and is a matter of debate. Their association is coincidence or one disease might be initiating the other. Immune deficiency because of carcinoma may welcome the entry of *Mycobacterium tuberculi,* or chronic damage to mucosa can lead to metaplasia followed with dysplasia and carcinoma. So, a large number of studies should be done emphasizing the relation between these two pathologies.
